# Atrial arrhythmias with mediastinal lymphadenopathy presentation of isolated atrial myocarditis

**DOI:** 10.1002/joa3.13206

**Published:** 2024-12-25

**Authors:** Muneeb Khawar, Mirza Muhammad Hadeed Khawar, Hannan Saeed

**Affiliations:** ^1^ Department of Cardiology King Edward Medical University Lahore Pakistan; ^2^ Punjab Institute of Cardiology Lahore Pakistan

Dear Editor,

We have read with great interest the article by Kumar et al., titled “Atrial Arrhythmias with Mediastinal Lymphadenopathy: Presentation of Isolated Atrial Myocarditis,” published in *Journal of Arrhythmia* (2024). The work provides valuable insights into the relationship between atrial arrhythmias and isolated atrial myocarditis (AM), emphasizing the relevance of this connection in young patients without conventional risk factors. The authors are to be commended for their efforts in addressing the diagnostic and therapeutic challenges posed by this condition. They aptly conclude that granulomatous myocarditis caused by sarcoidosis or tuberculosis should be considered the primary cause of atrial inflammation and its subsequent role in arrhythmogenesis.

The study employs a comprehensive approach to diagnosing AM in patients with unexplained atrial arrhythmias, utilizing a combination of histopathological examination and ^18^F‐FDG PET/CT. This methodology proves invaluable in distinguishing AM from other inflammatory and structural heart diseases. A particular strength of this work is the clear correlation established between imaging findings and clinical or histopathological data. The authors effectively employ a diagnostic algorithm that integrates atrial imaging, biopsy, and clinical assessment, thereby offering a systematic framework for diagnosis.

Another noteworthy aspect of the study is the emphasis on individualized treatment plans. Immunosuppressive therapy, including corticosteroids and methotrexate for patients with sarcoidosis, as well as anti‐tuberculous regimens for those with *Mycobacterium tuberculosis*, demonstrated significant clinical benefits. These interventions led to improvements in functional class, reductions in inflammatory markers, and the reversal of abnormal imaging findings, highlighting the therapeutic potential of these strategies. Furthermore, the authors' insights into the management of anticoagulation therapy for stroke prevention in patients with atrial arrhythmias are particularly relevant, as 26.7% of the patients in the study presented with ischemic strokes. This underscores the importance of vigilant monitoring and tailored management in this patient population.

Kumar et al. also highlights the potential of AM to serve as an independent substrate for atrial arrhythmias, even in the absence of common risk factors. This observation aligns with previous studies suggesting that inflammation, particularly granulomatous inflammation, can interfere with atrial electrophysiological properties and promote arrhythmogenesis.^1^, ^2^ Granulomatous infiltration has been shown to remodel atrial tissue, leading to electrical disturbances and an increased thromboembolic risk.^3^ Kumar et al.'s work reinforces these findings and provides clinical data that support the inflammatory hypothesis of arrhythmogenesis.

While the study offers significant contributions, there are some aspects that merit further discussion. The cohort was derived from a region endemic for tuberculosis and sarcoidosis, which raises questions about the generalizability of these findings to non‐endemic areas. Future studies involving more diverse populations could provide a broader understanding of AM's epidemiology. Additionally, while the median follow‐up of 26 months is reasonable, longer‐term follow‐up would provide more insight into arrhythmia recurrence, the progression of inflammation, and the long‐term cardiovascular outcomes. The lack of serological examinations for viral infections and anti‐myosin antibodies is another limitation, as these could have provided further insight into non‐granulomatous etiologies of AM.

The study also opens the door to the investigation of new therapeutic approaches. More targeted therapies, such as cytokine inhibitors or other immunomodulatory agents, may offer superior outcomes compared to traditional steroid‐based management. Moreover, novel imaging techniques, such as cardiac MRI with advanced sequences, could prove valuable in detecting atrial involvement in AM.^4^ These advancements could enhance diagnostic specificity and aid in distinguishing AM from other atrial disorders.

From a clinical perspective, this study serves as a timely reminder for clinicians to maintain a high degree of suspicion for AM in young patients with unexplained atrial arrhythmias, particularly in those with ischemic stroke. The application of advanced diagnostic technologies, such as ^18^F‐FDG PET/CT, may significantly improve detection rates when incorporated into the diagnostic pathway. Early diagnosis and the initiation of disease‐specific treatments, as emphasized by the authors, hold the potential to improve outcomes for affected patients.

We would like to thank the authors for their valuable contribution to the growing literature on atrial myocarditis. Their work not only enhances our understanding of the pathophysiology of inflammation, arrhythmias, and thromboembolism but also provides practical guidance for both diagnosis and treatment. We hope that their findings will inspire further research into the diverse causes and treatment strategies for AM.

Sincerely,

Muneeb Khawar

## CONFLICT OF INTEREST STATEMENT

The authors declare no conflict of interests for this article.

## ETHICAL APPROVAL

Ethical approval was not required for this study.
